# Comparative Toxicity of Fumigants and a Phosphine Synergist Using a Novel Containment Chamber for the Safe Generation of Concentrated Phosphine Gas

**DOI:** 10.1371/journal.pone.0000130

**Published:** 2006-12-27

**Authors:** Nicholas Valmas, Paul R. Ebert

**Affiliations:** 1 School of Molecular and Microbial Sciences, University of Queensland, St Lucia, Queensland, Australia; 2 School of Integrative Biology, University of Queensland, St Lucia, Queensland, Australia; The Research Institute for Children, United States of America

## Abstract

**Background:**

With the phasing out of ozone-depleting substances in accordance with the United Nations Montreal Protocol, phosphine remains as the only economically viable fumigant for widespread use. However the development of high-level resistance in several pest insects threatens the future usage of phosphine; yet research into phosphine resistance mechanisms has been limited due to the potential for human poisoning in enclosed laboratory environments.

**Principal Findings:**

Here we describe a custom-designed chamber for safely containing phosphine gas generated from aluminium phosphide tablets. In an improvement on previous generation systems, this chamber can be completely sealed to control the escape of phosphine. The device has been utilised in a screening program with *C. elegans* that has identified a phosphine synergist, and quantified the efficacy of a new fumigant against that of phosphine. The phosphine-induced mortality at 20°C has been determined with an LC_50_ of 732 ppm. This result was contrasted with the efficacy of a potential new botanical pesticide dimethyl disulphide, which for a 24 hour exposure at 20°C is 600 times more potent than phosphine (LC_50_ 1.24 ppm). We also found that co-administration of the glutathione depletor diethyl maleate (DEM) with a sublethal dose of phosphine (70 ppm, <LC_5_), results in a doubling of mortality in *C. elegans* relative to DEM alone.

**Conclusions:**

The prohibitive danger associated with the generation, containment, and use of phosphine in a laboratory environment has now been substantially reduced by the implementation of our novel gas generation chamber. We have also identified a novel phosphine synergist, the glutathione depletor DEM, suggesting an effective pathway to be targeted in future synergist research; as well as quantifying the efficacy of a potential alternative to phosphine, dimethyl disulphide.

## Introduction

The fumigant phosphine (PH_3_) is widely used in stored product protection owing largely to its potency, ease of use, and low cost. With the phasing out of ozone-depleting substances in accordance with the United Nations Montreal Protocol, phosphine has become the only economically viable fumigant for the protection of stored grain. The long-term use of phosphine is now under threat however, due to the development of high-level resistance in pest insects. This scenario has motivated efforts to determine the genetic basis of resistance [1]–[2] and to identify the genes that are responsible [3].

Despite decades of study and use, the precise mechanism of phosphine toxicity has not been determined. Toxicity is reliant on molecular oxygen [4]–[6] and is hypothesised to result from inhibition of the mitochondrial complex IV enzyme cytochrome *c* oxidase, resulting in production of reactive oxygen species and cellular oxidative stress [7]–[11].

Toxicological studies in mammals have shown that glutathione (GSH) provides important protection against phosphine induced disruptions to cells as GSH levels were found to decrease in rat tissue and human blood following phosphine exposure [12]–[15]. Conversely, addition of GSH to mouse cells partially protected them against phosphine-induced cell death, reactive oxygen species, and DNA damage [16]. The GSH depletor buthionine sulfoximine, which irreversibly inhibits γ-glutamylcysteine synthetase at the first step of GSH synthesis, can further lower the already reduced GSH levels in rats exposed to phosphine [15], although the effect on mortality of co-treatment with phosphine and buthionine sulfoximine was not determined. Furthermore, GSH depletion is reported to have no effect on phosphine induced mortality in insects [17].

A practical necessity to prepare for the potential loss of phosphine is identifying a replacement compound. For instance, the botanical insecticide dimethyl disulphide (DMDS) has been suggested as a grain fumigant and is being actively investigated as a soil disinfestant [18]–[19]. Coincidentally, the mode of action of DMDS has recently been proposed to be similar to that of phosphine, in that DMDS treatment results in inhibition of cytochrome *c* oxidase [20].

The nematode, *Caenorhabditis elegans* has not been widely used as a model organism for studies into fumigant toxicology, despite being ideal in many regards. As a nematode, it represents the class of organism that is the primary target of soil fumigation. It is easy to rear in the laboratory, with a generation time of three days, and due to tremendous reproductive capacity and small size it facilitates rapid screening of compounds for toxicity as well as accurate quantitative analysis. A simple but important practical matter for research purposes is that simultaneous, quantitative application of gaseous and soluble compounds is straightforward with *C. elegans*, whereas it is comparatively difficult with grain-feeding insects. The availability of phosphine resistant mutants [6] and a comprehensive suite of genomic resources (http://www.wormbase.org/) augment the value of *C. elegans* for phosphine resistance research.

Despite the need for phosphine research and the utility of research tools being developed in basic science laboratories, increasingly stringent occupational health and safety regulations in Australia, and likely elsewhere in the world, restrict the use of standard phosphine generation protocols. Such protocols are deemed unsafe in an academic research setting in which facilities are shared by large numbers of student trainees in laboratories often lacking adequate fail-safe systems in the event of power or equipment failure. The problem is compounded by the very high concentrations of gas required to treat organisms that are becoming increasingly resistant to phosphine exposure.

The following report describes a unique device designed specifically for the safe generation of phosphine gas from metal phosphide tablets within an enclosed laboratory, and also demonstrates the use of the soil nematode *C. elegans* as a tool for screening bioactive chemicals. The toxicity of the potential soil fumigant dimethyl disulphide was tested relative to phosphine, using the *C. elegans* system to obtain dose-response curves to the cytochrome *c* oxidase inhibitor, and also to look at potential synergism between phosphine and glutathione depletors.

## Methods

### Nematode Culture

Nematodes were grown at 20°C on NGM agar plates (3 g NaCl; 2.5 g peptone; 20 g agar; in 975 mL deionised water, autoclave then add 1mL of 5mg/L cholesterol in ethanol; 1 mL 1 M CaCl_2_; 1 mL 1 M MgSO_4_; 25 mL 1M potassium phosphate pH 6). Media containing 2% agar was used rather than the traditional 1.7%, to reduce the incidence of individuals burrowing into the media. Food was provided as a bacterial slurry of *E. coli* OP50 in deionised water.

Nematode eggs were obtained by treating breeding adults with a freshly prepared bleach solution (0.75 N NaOH; 1.5% NaOCl) for 5 minutes, and then rinsing 3 times with M9 buffer (6 g/L Na_2_HPO_4_; 3 g/L KH_2_PO_4_; 5 g/L NaCl; 0.25g/L MgSO_4_ •7H_2_O). Eggs were left to hatch overnight on an orbital shaker whilst suspended in M9 buffer. Synchronised populations of nematodes were produced by placing newly hatched larvae on NGM agar plates seeded with OP50, at which point they were deemed to be zero hours old.

### Chemical Treatment Conditions

Synchronised populations of nematodes were chemically treated when they were 48 hours old. One hour prior to treatment, nematodes were washed off their culture dishes using deionised water and transferred to 12-well tissue culture plates, which contained 2.5 mL of NGM agar per well and were pre-seeded with 20 µL of a 1∶30 dilution of OP50 slurry. The number of nematodes per well was then recorded and the plates were left to dry thoroughly before treatment. A dilution of at least 9 individuals per well was desired, in order to amass around 100 nematodes per plate.

Nematodes were chemically treated for 24 hours in glass desiccators that were sealed gas-tight using a rubber O-ring and clamps. Desiccators possessed screw thread adaptors sealed with silicon septa through which phosphine could be injected. After treatment and recovery time, the number of surviving individuals was determined by flooding the culture dish with M9 buffer and observing how many nematodes were freely moving in the aqueous environment. Exposure to either phosphine or DMDS has a narcotic effect on individuals that inhibits development and leaves them paralysed immediately after treatment, making it difficult to score the number of survivors by a motility assay. Therefore, nematodes were left to recover for up to 48 hours before being scored, the exact time being dependant on how quickly the recovering individuals started to produce progeny, which would complicate counting. In situations where there was a compound added to the agar which may affect the nematodes during the recovery period, it was made sure that the air control plates were counted at the same time as the phosphine plates.

### Phosphine Generation

**Figure 1:**
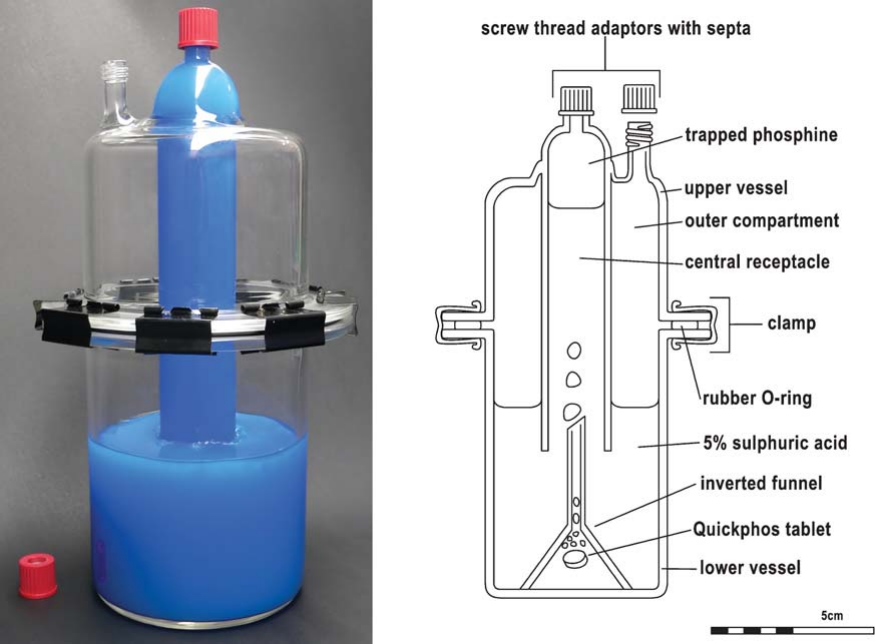
Phosphine Gas Generation Chamber A photograph of the gas generation chamber is shown (A) with blue liquid representing the sulphuric acid solution; as well as a schematic (B) labelled with the components of the system.

Gaseous phosphine was generated by dissolving aluminium phosphide tablets in a sulphuric acid solution and capturing the evolving gas. This procedure was performed in a chamber designed specifically for phosphine production and was located within a fume cupboard to minimize any risk of the gas escaping. The device is shown in figure 1 and consists of two glass vessels with ground flanges around the open ends which allow them to be secured together with a gas-tight seal using a rubber O-ring and clamps. The upper vessel consists of an inner gas receptacle which collects the trapped phosphine; and an outer compartment containing air which is displaced by the production of phosphine and which can be sealed off in the event that the fume cupboard ceases to function. The bottom vessel contains a reservoir of sulphuric acid solution which acts as both a barrier between the phosphine and external environment; as well as catalysing the generation of phosphine from aluminium phosphide tablets. To generate phosphine, the lower vessel is filled with approximately 1 L of 5% sulphuric acid and then a fragment of a Quickphos aluminium phosphide tablet (Bayer CropScience) was dropped into it. An inverted glass funnel was then positioned over the tablet which would trap and channel the gas through the neck and into the central receptacle of the upper vessel. A rubber O-ring was then positioned on the flanges of the lower vessel and the upper vessel was placed on top such that the central receptacle was directly over the funnel neck; and the O-ring was sandwiched between the flanges of the upper and lower vessels. A screw thread adaptor containing a silicon septum was used to seal the inner receptacle, and eight clamps were used to fasten the flanges of the vessels, and create a gas-tight seal. The air trapped within the central receptacle was then completely removed using a syringe, and the device left with the outer compartment remaining unsealed in a fume cupboard whilst the phosphine was produced.

### Phosphine Quantification

In addition to aluminium phosphide, Quickphos tablets also contain ammonium carbamate, which prevents phosphine from combusting by decomposing to ammonia and carbon dioxide. As the phosphine generated from Quickphos tablets is not pure, it had to be quantified before use. This was done by injecting a known volume into gas-tight glass desiccator (1 mL into 3 L) and measuring the resulting concentration; which could then be used to determine the concentration of the original gas stock. A continuous flow circuit was established by attaching the glass desiccator to a SmarTox-O gas monitor which had been pre-calibrated on pure phosphine, (The Canary Company Pty Ltd) and recording the phosphine concentration after 5 minutes at which time the mixture was homogenous.

### Chemical Administration

Nematode populations were exposed to phosphine by sealing them in gas-tight desiccators and injecting the desired amount of phosphine. Dimethyl disulphide is a volatile liquid at room temperature and was administered by dispensing the required volume onto a glass Petri dish which was sealed inside the desiccator with the nematodes. The volumes of DMDS used in this study evaporate within a few minutes. dl-Buthionine-[S,R]-sulfoximine (BSO) and diethyl maleate (DEM) were added directly to warm NGM agar as the culture plates were being prepared; BSO having been dissolved into deionised water first, while DEM, a liquid at room temperature, was directly added. All NGM agar plates were freshly made within 1 day of being used and were stored in the dark at 4°C to minimised chemical degradation.

### Statistical Analysis

Genstat 7 (VSN International Ltd) was used to perform all statistical calculations. Phosphine generation data was analysed using linear regression whilst dose-response data was subject to probit regression. Mortality values for individual biological replicates were adjusted using Abbott's formula, after which the data was pooled for further analysis. Six transformations were performed on the data (probit, logit and complementary log-log on the response variate; and linear and logarithm on the explanatory variate) and regression was performed. The transformation which produced the smallest residual deviance was used to approximate the does-response relationship.

## Results and Discussion

### Phosphine Generation

**Figure 2:**
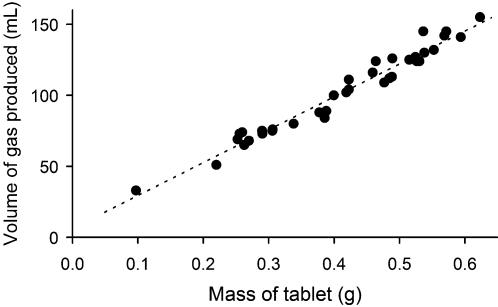
Amount of Gas Produced by Quickphos Fragments The relationship between the mass of a fragment of Quickphos aluminium phosphide, and the volume of gas it produces in the chamber shown in figure 1 has been approximated using linear regression. Regression statistics are as follows: slope = 232.48; intercept = 5.99; r^2^ = 0.963; n = 34.

The phosphine generation protocol, which includes the use of a custom designed containment chamber, allows for safe generation of phosphine gas within a laboratory environment. The amount of gas produced by Quickphos tablet fragments of various masses is shown in figure 2, and by using linear regression the relationship between mass and gas volume has been approximated to 232 mL/g. The time taken for a tablet fragment to completely dissolve is dependent on both the mass as well as the shape of the fragment, and due to a lack of consistency in fragment shape it was not possible to establish a relationship between mass and time taken to dissolve. Of all the fragments used in this study however, none took longer than 3½ hours to completely dissolve. The central receptacle of the vessel shown in figure 1a can contain approximately 150 mL of gas, beyond which it will be released into the outer compartment of the upper vessel. Thus, the unit can easily contain the gas produced from 0.65 grams of a Quickphos tablet. Results for chemically pure aluminium phosphide will differ somewhat, because the commercial Quickphos formulation contains not only aluminium phosphide, but also ammonium carbamate.

The standard phosphine generation protocol uses a device similar to that presented in figure 1 except for the absence of the upper vessel, and consequently the outer chamber which can contain leaking phosphine gas. The standard device did not pass Australian Occupational Health and Safety requirements due to a significant leakage of phosphine from the central gas collection receptacle, through the liquid bath and into the surrounding air. The rate of leakage is low due to the low solubility of phosphine in aqueous solutions, but in the vicinity of the bath, the phosphine concentration reached 1 ppm, which exceeded the permissible exposure limit (PEL) of 0.3 ppm. This was deemed to be an unacceptable risk in the event of a failure of the laboratory ventilation system.

The new chamber includes an upper vessel with a screw thread adaptor that enables the chamber to be completely sealed in the event of a fume cupboard malfunction, thereby preventing any gas escape. We generally allow the gas to be generated completely prior to sealing the septum. In this way, we avoid the pressure build up that otherwise occurs due to gas generation in a restricted volume. It is also possible, however, to carry out the entire phosphine generation process while the chamber is sealed, as there is approximately 800 mL of air above the aqueous bath which acts as a pressure buffer. The chamber was stress-tested to determine its ability to withstand pressure by injecting a large volume of air into the sealed chamber through the septum above the gas collecting chamber. It was found that the chamber could safely contain up to 300 mL of gas. Further addition of gas breached the gas-tight seal by blowing out the O-rings from the flanges of the vessel.

### Phosphine and Dimethyl Disulphide Toxicity

**Figure 3:**
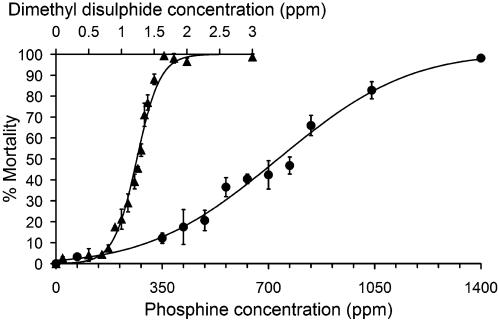
Phosphine and Dimethyl Disulphide Mortality of N2 at 20°C Mortality of wild-type (N2) *C. elegans* when exposed at 20°C for 24 hours to phosphine (•) and dimethyl disulphide (▴). Regression lines are based on probit/linear and logit/linear relationships respectively. Data points are weighted means from biological replicates±weighted SEM. The LC_50_ of phosphine at 20°C for N2 is 732 ppm; and for DMDS is 1.24 ppm. Plates were counted as follows: 0 ppm phosphine and 0 ppm to 1.1 ppm DMDS, immediately after fumigation; 70 ppm phosphine and 1.2 ppm to 1.5 ppm DMDS, after 24 hours recovery; doses above 70 ppm phosphine and 1.5 ppm DMDS, after 48 hours recovery.

The wild-type *C. elegans* line N2 was exposed to a range of phosphine concentrations and the mortality calculated (figure 3). Probit/linear regression estimates that the LC_50_ for a 24 hour fumigation period for this line at 20°C is 732 ppm (95% CI: 708 to 757 ppm). This value is 4 times that previously reported for the LC_50_ of a 24 hour fumigation at 25°C, ∼185 ppm (0.26 mg/L) [6]. This is consistent with previous observations of a positive correlation between temperature and toxicity of phosphine in several insect species [21]–[28] and also in rats [29]. This result is explained as an increase in the uptake and metabolism of phosphine by the animals due to higher metabolic rates at higher temperatures [27].

The toxicity of DMDS toward *C. elegans* was determined following a 24 hour exposure at 20°C (figure 3). The LC_50_ is 1.24 ppm (95% CI: 1.20 to 1.27 ppm) as estimated using logit/linear regression. The LC_50_ for DMDS is less than 1/600^th^ that of phosphine. Thus, while the mechanism of action has been proposed to be similar between the two fumigants, DMDS is dramatically more toxic toward *C. elegans* than is phosphine. Whilst no other comparative toxicity studies have been carried out between phosphine and DMDS, independent experiments have been reported for the cowpea weevil *Callosobruchus maculatus* [20], [30]–[33]. Comparison of LC_50_ values for 24 hours treatments with each fumigant supports the general conclusion that DMDS is much more toxic than is phosphine; a fact made inconspicuous because the culture and fumigation conditions between the studies were not identical. It does appear that the difference in toxicity between the two compounds is about an order of magnitude greater in the nematode, *C. elegans* than in the insect, *C. maculatus.* As the physical properties of DMDS make it most suitable as a soil fumigant for which nematodes are the primary target, its toxicity toward nematodes is a prime consideration. The extreme toxicity of this compound as well as the ability of the human nose to detect concentrations of this chemical well below the allowable exposure limits bode well for the efficacy and safety of DMDS as a soil fumigant.

### Phosphine and Diethyl Maleate Synergism

**Figure 4:**
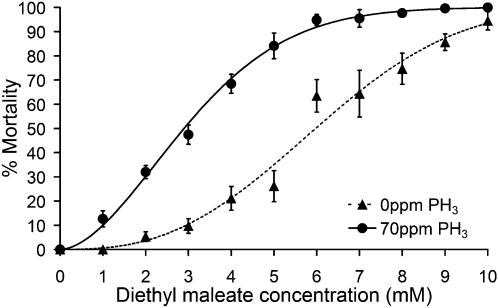
Diethyl Maleate Interaction with Phosphine Mortality of wild-type (N2) *C. elegans* when exposed to diethyl maleate and two different doses of phosphine: 0 ppm (▴); and 70 ppm (•); at 20°C for 24 hours. Regressions lines are based on complementary log-log/log relationships, and data points are weighted means from biological replicates±weighted SEM. The LC_50_ of DEM in the absence of phosphine at 20°C for N2 is 5.98 mM; and with 70 ppm PH_3_ is 2.9 mM. All plates were counted after 24 hours recovery.

A sub-lethal dose of phosphine (70 ppm) was tested together with the GSH depletors DEM and BSO. At the concentrations tested (1 µM, 10 µM, 100 µM, 1 mM, 10 mM) BSO failed to cause any mortality either by itself, or in combination with phosphine (data not shown). Lethal doses of BSO could not be achieved as it was not practical to dissolve BSO in the growth medium at concentrations greater than 10 mM. It is likely that the high tolerance of *C. elegans* to BSO is due to the hydrophilic character of the compound which likely prevents it from penetrating the hydrophobic cuticle of the nematodes. Using the more hydrophobic GSH depletor diethyl maleate, it was possible to induce mortality in *C. elegans* (figure 4). The LC_50_ following 48 hour exposure at 20°C was determined to be 5.98 mM (95% CI: 5.634 to 6.3 mM) by complementary log-log/log regression. A constant, sub-lethal concentration of phosphine (70 ppm) caused a synergistic doubling in mortality due to exposure to DEM, with an LC_50_ of 2.896 mM (95% CI 2.719 to 3.063 mM). This is the first report of phosphine and a glutathione depletor acting synergistically to increase mortality, as a previous study [17] reported no change in phosphine susceptibility in insects treated with BSO.

### Conclusion

In the present study we describe a unique phosphine generation chamber that allows for the safe production and containment of the gas in a laboratory environment. We use this device and the model organism *C. elegans*, as part of a screening protocol for the assessment of chemical toxicity relative to, and in conjunction with, phosphine. The toxicity of dimethyl disulphide supports its development as a soil fumigant. Co-treatment with phosphine and diethyl maleate identified for the first time, a protective mechanism against phosphine exposure in invertebrates that had previously been observed in mammals. It is hoped that improved handling of the poisonous gas will encourage research on the fumigant, especially with novel research strategies in academic research labs, so that more may be understood about the pathways of toxicity and resistance.
